# Demonstration of Alpha-Band Entrainment via Low-Field Magnetic Stimulation: A Simulation-Driven Proof of Concept

**DOI:** 10.3390/bioengineering13040395

**Published:** 2026-03-29

**Authors:** Costin Dămășaru, Georgiana Roșu, Leontin Tuță, Alexandra Cernian, Mihaela Rus

**Affiliations:** 1 Faculty of Applied Sciences, National University of Science and Technology Politehnica Bucharest, 060042 Bucharest, Romania ; costin.damasaru@gmail.com; 2 Veruvis Research Center, 011772 Bucharest, Romania; 3 Faculty of Communications and Electronic Systems for Defence and Security, Ferdinand I Military Technical Academy, 050141 Bucharest, Romania; 4 Faculty of Automatic Control and Computers, National University of Science and Technology Politehnica Bucharest, 060042 Bucharest, Romania; alexandra.cernian@upb.ro; 5 Faculty of Law and Administrative Sciences, Ovidius University, 900470, Constanța, Romania; mihaela.rus@365.univ-ovidius.ro

**Keywords:** brainwave entrainment, figure-of-eight coil, neural oscillations, neuromodulation

## Abstract

Low-field magnetic stimulation (LFMS) has been proposed as a non-invasive approach for modulating cortical oscillations through electromagnetic coupling. Frequency-aligned enhancement of alpha-band activity is of interest due to its association with cortical inhibitory balance and relaxed wakefulness. This study investigates whether a 10 Hz LFMS applied to the occipital area can induce measurable alpha-band modulation. Electromagnetic simulations were performed to determine magnetic flux distributions within a simplified spherical head model with magnetic susceptibility, which was approximating the brain’s parameters. The 10 Hz stimulation waveform—a positive ramp sawtooth—was analyzed in both time and frequency domains. Electroencephalographic (EEG) recordings were obtained before and after stimulation, and spectral analyses of relevant occipital channels were used to quantify the power redistributions. Simulations indicated localized magnetic field gradients in the occipital region. Post-stimulation EEG recordings showed a redistribution of spectral power toward the alpha-band, representing approximately 50% of total occipital spectral power, with relative increases exceeding 140% across the analyzed channels. These combined modeling and electrophysiological findings provide preliminary proof-of-concept evidence that frequency-aligned LFMS is associated with a redistribution of spectral power toward the alpha-band.

## 1. Introduction

Brainwaves or neural oscillations represent the electrical patterns of neural activity and reflect the coordination between neuronal populations located separately [1]. These oscillations are typically grouped into several frequency bands—delta (0.5–4 Hz), theta (4–8 Hz), alpha (8–12 Hz), beta (12–30 Hz), and gamma (30–100 Hz)—and each category has been associated with characteristic physiological and cognitive states [2,3]. For instance, posterior alpha rhythms are enhanced during the state of relaxed wakefulness, and they are known to control sensory gating and cortical inhibitory balance [4]. Since stress and anxiety states have been linked to alpha activity alterations, research strategies were developed to modulate these frequencies [5].

Brainwave entrainment refers to the brain’s natural ability to synchronize the rhythm of its endogenous neural oscillations with the one imposed by an external stimulus [6,7]. While the mechanisms of auditory and visual entrainment have been documented intensively [8,9], electromagnetic stimulation provides a more complex mechanism and a more direct physical interface with the neural tissue [10,11]. Time-varying magnetic fields induce electric fields in conductive media according to Faraday’s law of induction, which is as follows: ∇×E=−∂B/∂t. These induced electric fields can influence membrane polarization and network synchronization depending on their magnitude, spatial distribution, and temporal structure [12,13,14]. In conventional transcranial magnetic stimulation (TMS), time-varying magnetic fields generate electric fields strong enough to evoke neuronal depolarization [15,16,17]. Low-field magnetic stimulation (LFMS) operates at significantly lower amplitudes; current research indicates that it relates to neural dynamics through subthreshold mechanisms, influencing the oscillatory activity without directly triggering action potentials [18,19].

The capacity of weak electric fields to influence neural networks is still under active debate, with one perspective stating that electric field magnitudes below established depolarization thresholds are unlikely to exert meaningful biological effects [20,21]. On the other hand, it is proposed that even small fields, coherently aligned with intrinsic oscillations, may alter spike timing probability or network synchronization [18,22]. Experimental and computational studies of weak-field interactions suggest that network sensitivity exceeds the activation threshold of a single neuron [23].

Besides field magnitude, its spatial distribution is a major factor of neuromodulation efficacy. The coil geometry determines the magnetic flux gradients and, consequently, the induced field within tissue [24]. High-field TMS research has demonstrated a trade-off between penetration depth and focality, with figure-of-eight configurations providing a more localized targeting compared to circular coils [25,26,27]. However, optimization strategies developed for high-field stimulation cannot be directly extrapolated to low-field applications in the latter case, as the field gradients and temporal coherence may be more relevant than the peak amplitude.

Computational electromagnetic modeling offers a rigorous method for predicting magnetic flux density (***B*** field) and estimating induced electric field distributions within anatomical structures [28,29,30]. When combined with electroencephalographic (EEG) spectral analysis, it can help establish a correlation between the field configuration and measurable changes in cortical oscillatory activity [31]. Such integration is essential for evaluating the plausibility of proposed weak-field modulation mechanisms.

This study investigates whether simulation-guided low-field magnetic stimulation at 10 Hz can induce measurable alpha-band enhancement when applied to the occipital cortex, a region characterized by strong intrinsic alpha generators [32,33]. Two coil configurations were investigated: a typical commercial solenoid and a figure-of-eight coil developed with the scope of further enhancing the hypothesized effect. Electromagnetic simulations were conducted to characterize the magnetic flux distribution within a simplified spherical head model, which approximated brain magnetic susceptibility [34,35]. The stimulation waveform, a 10 Hz positive ramp sawtooth, was analyzed in both time and frequency domains to assess its spectral content. The EEG recordings performed before and after the stimulation, for both investigated coils, were evaluated using the metric of band power redistribution and focused on the occipital channels.

The main observation is a substantial shift in the spectral power toward the alpha-band in the post-stimulation measurements, for both coils. These findings provide proof of concept that frequency-aligned low-field magnetic stimulation can influence cortical oscillatory balance. Although further quantitative electric field modeling, anatomically realistic simulations, and controlled experimental validation are necessary, the study demonstrates the feasibility of integrating electromagnetic field theory and electrophysiological measurement within applied neuromodulation engineering research.

The paper is structured as follows: Section 2 details the research methodology, including (Section 2.1) a description of the commercial device, (Section 2.2) specifications of the electromagnetic transducer and its optimized variant tailored for occipital stimulation, (Section 2.3) the excitation signals used during the entrainment process, and (Section 2.4) the experimental protocol and data analysis procedures. Section 3 presents results obtained for both the original and optimized coils, divided into (Section 3.1) magnetic field simulations assessing focality and penetration depth, and (Section 3.2) EEG measurements evaluating modulation of alpha-band activity. Section 4 provides a comparative discussion of these results. Section 5 summarizes the main findings and conclusions.

## 2. Materials and Methods

### 2.1. Neuromodulation Device Description

**Figure 1:**
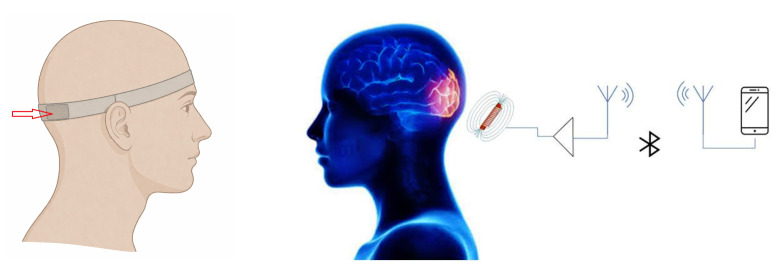
Headband positioning on a human head model, with the coil located in the Oz position, marked by a red arrow.

In this study, the external stimulus is delivered through a magnetic coil embedded within a headband which is positioned over the central occipital lobe and aligned with the position of the Oz electrode [36], as illustrated in Figure 1. The scope of this placement is to stimulate the cortico-limbic pathways connected to key neural structures. More precisely, the occipital cortex maintains reciprocal connections with limbic areas via occipitotemporal and other associative pathways, allowing sensory and visual inputs to affect emotional and stress-related processes.

Direct activation of subcortical structures by low-field magnetic stimulation remains limited. However, recent advances in stimulation mechanisms have demonstrated the modulation of deeper brain regions explained by a coordinated network response. Within the scope of this work, the focus is therefore on exploiting such network-driven mechanisms rather than on direct subcortical stimulation [22].

In addition to the headband, the device includes a software component in the form of a mobile application installed on a smartphone. The headband connects to the mobile device either via a standard audio jack or wirelessly through a Bluetooth adapter. The application hosts several stimulation programs tailored to specific outcomes, such as improving concentration, reducing anxiety, or enhancing sleep quality. Each program generates a stimulation signal at a frequency associated with the intended cognitive or emotional state [37].

### 2.2. Magnetic Stimulation Transducer—Cylindrical vs. Figure-of-Eight Designs

**Figure 2:**
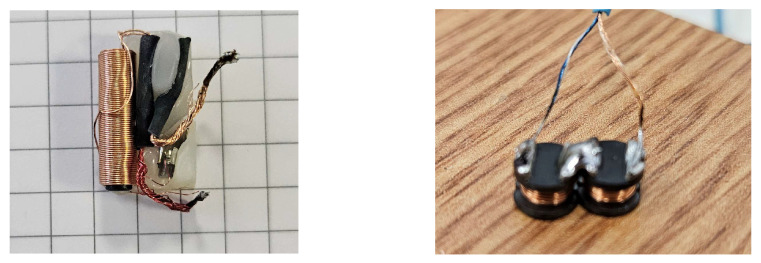
Magnetic transducer—original coil (**left side**) and optimized coil (**right side**).

The baseline magnetic stimulation transducer consists of a multi-layer cylindrical coil, as illustrated in Figure 2 (left). This coil comprises three layers of 70 turns each, resulting in a total winding count of *N* = 210. The copper wire has a diameter of approximately *ϕ* = 0.3 mm, and the solenoid incorporates a ferrite core with a relative magnetic permeability of *µ_r_* = 1000. The core dimensions are approximately *D* = 2.5 mm in diameter and *l* = 16 mm in height.

Drawing on prior work [25,26] that highlights the advantages of figure-of-eight coil geometries for focal stimulation, we developed an optimized transducer design. The proposed solution consists of two surface-mount device (SMD) coils with ferrite cores, arranged in a figure-of-eight configuration and connected in series, as illustrated in Figure 2 (right). Each SMD coil has a diameter of 9.8 mm and a height of 5.8 mm. The pair was selected to ensure that the overall geometrical dimensions closely matched those of the original cylindrical coil, while the series connection preserved comparable electrical parameters.

**Figure 3:**
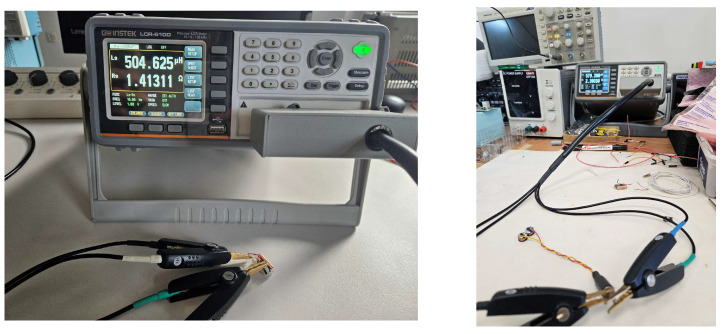
Measurement of the coil inductance—setup and result display.

Electrical characterization of both the cylindrical and figure-of-eight coils was performed using an LCR-6100 m (GW Instek, New Taipei City, Taiwan) with Kelvin connectors. The measurement setup is shown in Figure 3. Equivalent series inductance and resistance values, obtained at 10 Hz (corresponding to the alpha brainwave frequency), are summarized in Table 1.

### 2.3. Magnetic Stimulation of Brain Tissue

**Figure 4:**
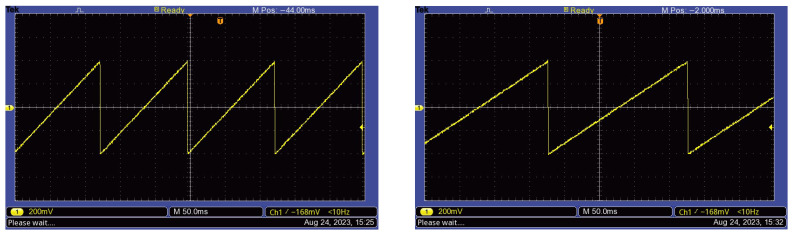
The sawtooth signal for: the 8 Hz anti-anxiety program (**left side**) and the 5 Hz the improve-sleep program (**right side**).

To evaluate the functional performance of the neuromodulation device, we first analyzed the stimulation signals generated by the mobile application across several built-in programs, including anti-anxiety, meditation, improved sleep, and anti-stress. In the initial stage of testing, the device was operated in its original configuration using the cylindrical coil. Across all programs, the stimulation waveform consisted of a positive ramp sawtooth signal, with frequency as the sole variable parameter. Representative examples are shown in Figure 4, including the anti-anxiety program at 8 Hz (left) and the improved sleep program at 5 Hz (right), which were acquired using a TBS 1102B-EDU Digital Oscilloscope (Tektronix, Beaverton, OR, USA).

**Figure 5:**
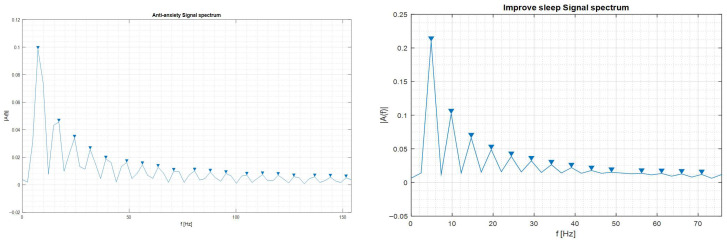
The spectrum of the sawtooth signal for: the 8 Hz anti-anxiety program (**left side**) and the 5 Hz improve-sleep program (**right side**).

Spectral analysis of these signals was performed using MATLAB2025a’s FFT algorithm. The resulting spectra, illustrated in Figure 5, demonstrate the presence of harmonics up to the 19th order for both the 8 Hz and 5 Hz programs.

**Figure 6:**
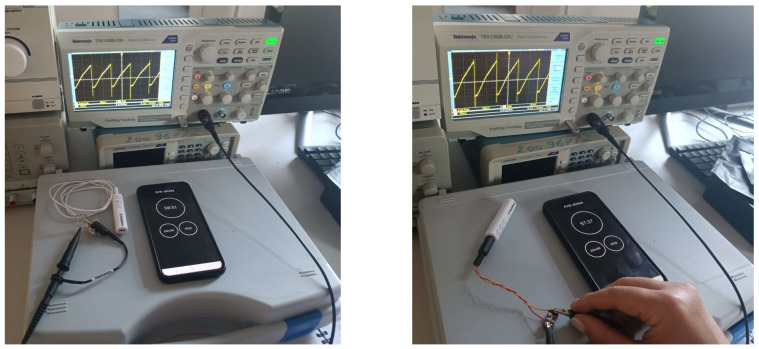
The measurement configuration and sawtooth signal generated within the 10 Hz anti-anxiety program, for the original coil configuration (**left side**) and the proposed coil configuration (**right side**).

Subsequently, we examined how coil design and impedance affect the signals delivered by the device. For this purpose, both the original cylindrical coil and the optimized figure-of-eight coil were connected via a standard audio jack. Since the focus of this study was the comparative evaluation of alpha-band entrainment, we selected the anti-anxiety program configured to generate a 10 Hz positive ramp sawtooth signal. The measured peak-to-peak voltages at the coil terminals were 380 mV for the cylindrical coil and 500 mV for the optimized coil, as shown in Figure 6.

### 2.4. Testing Protocol and Signal Processing

**Figure 7:**
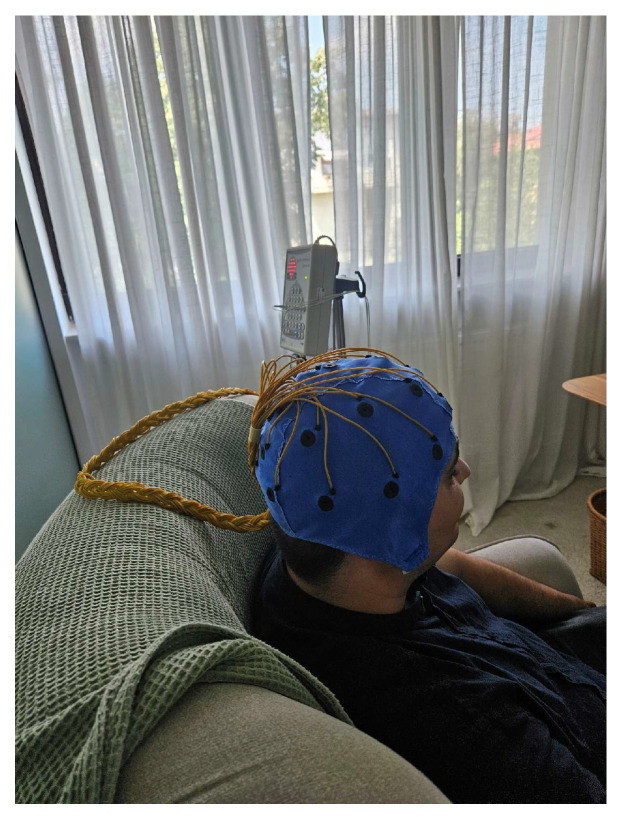
Signal acquisition using a 21-channel EEG cap and Neuro.

To evaluate the effectiveness of the neuromodulation device, electroencephalographic (EEG) recordings were performed before and after 10 Hz neurostimulation, as shown in Figure 7. Recordings were acquired from one of the authors using a 21-channel EEG setup over a 10 min duration. Between the two recording sessions, the neuromodulation stimulus was applied for 10 min to the occipital region of the participant’s head. During all sessions, the participant kept their eyes closed to minimize visual interference and reduce variability in occipital activity.

To avoid carryover effects, the experiments were conducted on consecutive days: the original cylindrical coil was tested on the first day, and the optimized figure-of-eight coil on the second. EEG acquisition was performed using the NEURON-SPECTRUM-4/P system (Neuromed, Entraigues-sur-la-Sorgue, France), and data visualization and initial analysis were conducted with NeuroGuide 2.9 software (Applied Neuroscience Inc., St. Petersburg, FL, USA).

**Figure 8:**
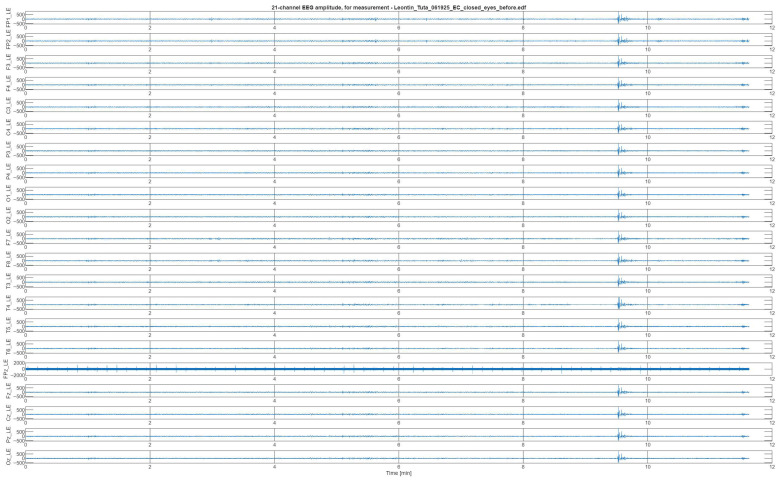
The 21-channel EEG signal amplitudes measured prior to neuro-stimulation treatment.

Figure 8 illustrates the raw EEG signals acquired from the 21 channels in one set of measurements. The sampling frequency was set at 500 Hz, resulting in *N* = 300,000 samples per channel for the 10 min sessions. For tests that slightly exceeded 10 min, datasets were truncated to ensure uniform length and facilitate alignment during processing. Since stimulation was applied at the occipital site, the O1_LE, O2_LE, and Oz_LE channels were selected for detailed analysis. The data was exported in .edf format and subsequently processed in MATLAB. Signal processing included filtering and segmentation procedures to compute spectrograms, as described in detail in Section 3.2.

## 3. Optimization Results

### 3.1. Magnetic Stimulation Transducer Analysis and Optimization

**Figure 9:**
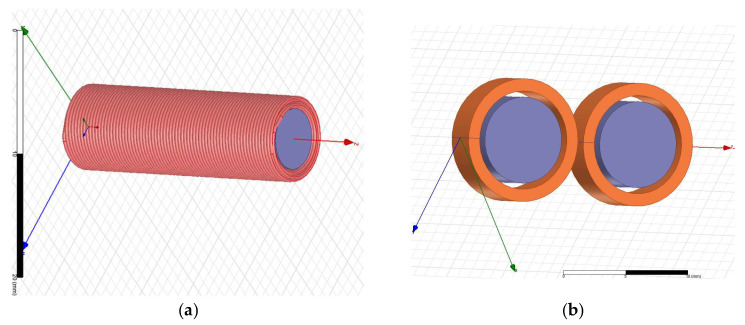
(**a**) Original cylindrical coil model; (**b**) optimized figure-of-eight coil model.

The comparative assessment of the original and optimized coils was first conducted in a simulation environment. Virtual models of both transducers were developed in Ansys Maxwell AEDT 2023 R2 (Ansys Inc., Canonsburg, PA, USA), as illustrated in Figure 9. These models reproduced the geometrical parameters of the physical coils while maintaining comparable overall spatial volumes.

The original cylindrical coil consisted of three layers of 70 turns each, with a wire diameter of approximately *ϕ* = 0.3 mm. The solenoid employed a ferrite core with a relative magnetic permeability of *µ_r_* = 1000 and approximate dimensions of *D* = 2.5 mm in diameter and *l* = 16 mm in height.

The optimized figure-of-eight coil was modeled as two cylindrical coils connected in series and separated by 0.5 mm. Each coil contained 10 turns of wire with a diameter of approximately *ϕ* = 0.3 mm. The ferrite cores had dimensions of *D1* = 10 mm in diameter and *l1* = 3 mm in height.

Following model construction, magnetic field distributions were simulated with each coil energized by a 1 A current. To approximate biological conditions, a simplified human head model was implemented as a sphere with a radius of 89 mm [17,18,19,20], filled with a liquid characterized by a magnetic susceptibility of *χ* = −6.928 × 10^−6^ [21,22]. This value corresponds to typical brain tissue susceptibility. Although simplified, this approach is consistent with established practices for modeling the electromagnetic properties of the brain.

This spherical model represents a simplified first-order approximation commonly used in preliminary electromagnetic field studies. It does not account for multilayer conductivity differences between the skull, cerebrospinal fluid (CSF), gray matter, and white matter, nor does it incorporate cortical folding geometry. Such simplifications may introduce field-strength estimation errors of up to 30% compared to anatomically realistic models [23]. The homogeneous approximation was deemed sufficient for this proof-of-concept study focused on comparative coil assessment and field distribution patterns, but future work will employ realistic multi-layer finite element method (FEM) head models for more accurate quantitative predictions.

To estimate the induced electric field magnitude, we applied Faraday’s law of induction. For the 10 Hz sawtooth waveform with a simulated peak magnetic field of approximately 0.5 mT, the time-varying component induces an electric field of approximately 0.2 V/m within cortical tissue. While this magnitude is well below the classical neuronal depolarization threshold, it falls within the range shown to influence spike timing and network synchronization in weak-field studies. For instance, in vitro work by Reato et al. [21] demonstrated that fields as low as 0.2 V/m produce measurable spike-phase entrainment, where spike timing becomes locked to the field’s rising edge. The temporal alignment of the 10 Hz stimulation with intrinsic occipital alpha generators may enable resonant amplification through network feedback mechanisms, allowing subthreshold perturbations to shift oscillatory balance without directly exciting individual neurons.

It must be noted that the induced electric field magnitude of 0.2 V/m is a model-dependent estimate based on a simplified homogeneous spherical phantom. As these models do not account for cortical folding or realistic tissue conductivity, this value represents a first-order approximation, with potential estimation errors of up to 30% compared to anatomically realistic finite element models. While the sawtooth waveform introduces harmonic components across multiple frequency bands due to its non-sinusoidal shape, its dominant spectral component remains centered at the fundamental frequency of 10 Hz. This approach provides frequency-aligned stimulation rather than strict frequency specificity, as the harmonic content may interact with neural oscillations beyond the alpha-band. However, the concentrated spectral energy at 10 Hz makes it suitable for targeting alpha-band resonance in the occipital cortex.

**Figure 10:**
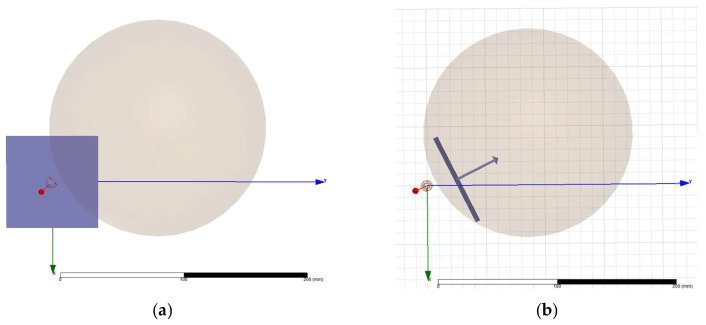
Field computation planes: (**a**) sagittal plane amid the coil; (**b**) occipital tilted plane.

Both coil models were positioned relative to the spherical head phantom in accordance with the physical placement of the device headband. Two cross-sectional planes were used to evaluate the resulting magnetic field distributions in the occipital region, where stimulation effects are expected (Figure 10): left panels—the XOY plane passing through the center of the coil (analogous to the sagittal plane of the head model); right panels—a tangent-aligned plane representing the cortical surface of the occipital lobe targeted by stimulation.

**Figure 11:**
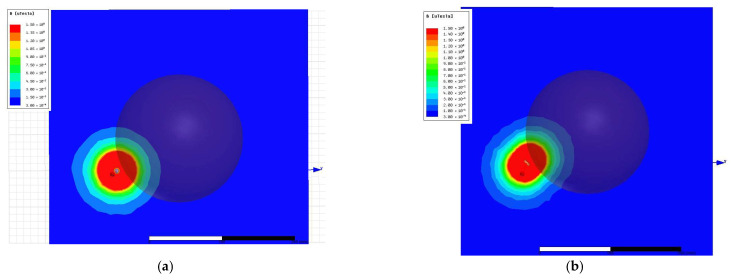
Magnetic field distribution in the sagittal plane (parallel to XOY) amid the coil. (**a**) Original cylindrical coil; (**b**) figure-of-eight coil.

**Figure 12:**
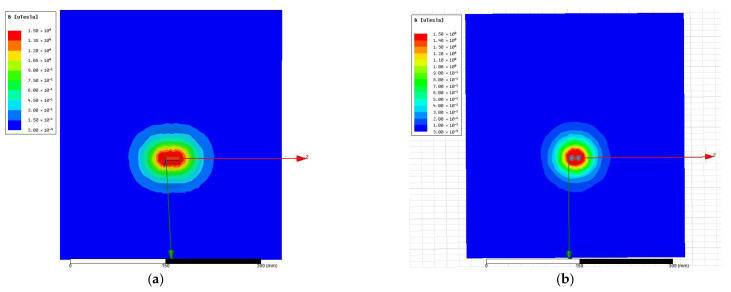
Magnetic field distribution in a tilted plane, parallel to the sphere tangent, with a section representing the targeted brain area. (**a**) Original cylindrical coil; (**b**) figure-of-eight coil.

For comparison, the magnetic field distributions generated by a 1 A current were computed for both coil designs across these planes. Results, presented in Figure 11 and Figure 12, were evaluated in terms of field penetration and focality. To enable direct comparison, identical magnetic field scales were applied in all visualizations.

Quantitative comparison of the two coil configurations was performed under identical simulation conditions (1 A excitation current). Measured peak-to-peak voltages at the coil terminals were 380 mV for the cylindrical coil and 500 mV for the figure-of-eight coil, yielding a raw voltage ratio of approximately 1.32. Accounting for the slightly larger inductance of the figure-of-eight configuration, the effective magnetic field amplitude was approximately 1.2× higher for the optimized coil. Analyses of the spatial field distributions revealed that the full width at half maximum (FWHM) of the high-field region decreased from approximately 12 mm (cylindrical) to approximately 7 mm (figure-of-eight), representing a ~40% reduction in focal width. The spatial gradient magnitude at the coil center increased from approximately 0.08 T/mm to approximately 0.10 T/mm, a ~30% enhancement in field gradient. Applying Faraday’s law, the temporal derivative of the magnetic field was approximately two times higher for the figure-of-eight coil, combining the voltage increase with the steeper spatial gradients inherent to the focused flux geometry.

### 3.2. Brainwave Entrainment EEG Results

The first stage of processing was aimed at artifact removal. In order to remove the 50 Hz power-line interference present in all EEG channels, including the three occipital channels of interest (O1_LE, O2_LE, and Oz_LE), a stopband finite impulse response (FIR) filter was implemented. The filter was designed with an order of *L* = 254 and stopband cutoff frequencies at 47 Hz and 53 Hz. The chosen order represented a compromise between computational efficiency and the sharpness of the bandwidth filter characteristic.

The inherent delay of *L*/2 = 127 samples introduced by the FIR filter is negligible relative to the total sample size *N* of each channel or of any selected time segment used for spectrogram analysis.

**Figure 13:**
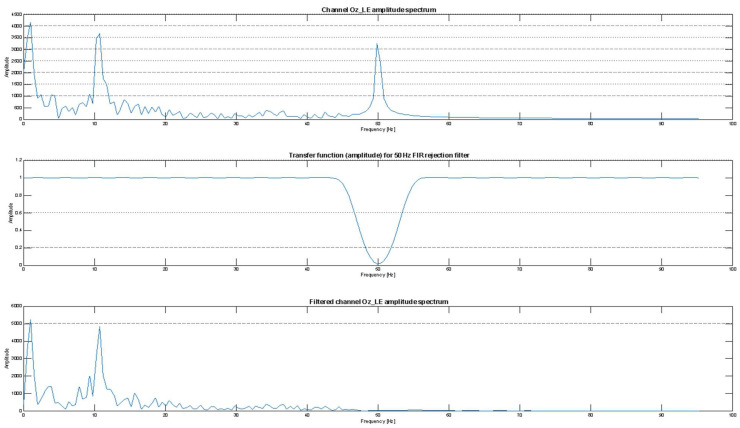
Representative example for the artifact removal process—the Oz channel spectrum before (**upper side**) and after (**lower side**) applying the FIR stopband filter (**middle graph**).

The effectiveness of artifact removal is illustrated in Figure 13, where the example represents the figure-of-eight post-treatment measurement of the Oz channel. The upper panel shows the FFT spectrum of the Oz channel prior to filtering, where a prominent 50 Hz component is visible. The middle panel presents the amplitude response of the designed filter while the lower panel depicts the spectrum after filtering, confirming suppression of the artifact.

**Figure 14:**
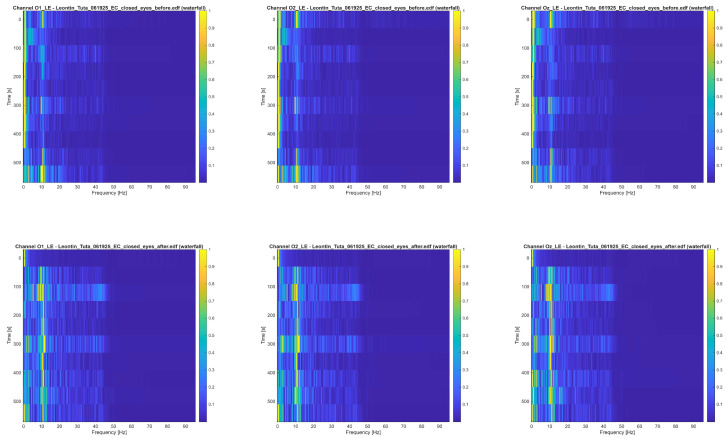
Waterfall representations of occipital channels O1 (**left**), O2 (**middle**), Oz (**right**): before the treatment (**upper side**) and after the treatment (**lower side**).

To assess the impact of the 10 min neuromodulation program, spectrograms were generated from EEG signals recorded before and after stimulation. The occipital channels were segmented into 1 min intervals, and for each segment the Discrete Fourier Transform (DFT) was computed according to the following:

(1) $$X_{i,s}\left[k\right]=\sum_{n=0}^{N-1}x_{i,s}\left[n\right]\cdot e^{-j\frac{2\pi }{M}kn},k=0..M-1$$

where *x_i,j_*[*n*] are the EEG time segments, *i* ∈ {O1_LE, O2_LE, Oz_LE} is the channel index, *s* is the segment index, and *M* = 1024 is the DFT size or number of points in the frequency domain. Figure 14 presents the resulting waterfall plots. The upper set of graphs correspond to pre-stimulation recordings, while the lower set correspond to post-stimulation with the figure-of-eight coil. Within each set, the O1_LE, O2_LE, and Oz_LE channels are displayed in the left, middle, and right columns, respectively.

For quantitative evaluation, the signal power was computed across the EEG frequency bands, with particular emphasis on the alpha-band (8–12 Hz) as the stimulation protocol was tuned to 10 Hz. The percentage distribution of power across bands, normalized according to Relation (2), is provided in Table 2 and Table 3 for pre- and post-treatment signals.

(2) $$P=\frac{1}{M}\sum_{k}{\left|X\left[k\right]\right|}^{2}$$

To facilitate comparison, relative changes in band power were calculated according to the following:

(3) $$R=\frac{After-Before}{Before}\cdot 100$$

As summarized in Table 4, the alpha-band exhibited the most pronounced increase across all occipital channels, with a maximum relative increase exceeding 200% at Oz_LE. Following stimulation, approximately 50% of the total signal power was concentrated within the alpha-band. This marked enhancement in alpha activity supports the effectiveness of the developed neuromodulation device in promoting power redistribution towards the targeted frequency.

The observed alpha enhancement represents a redistribution of relative power rather than a global amplification of total EEG activity. Band powers were normalized to the total signal power within each condition (pre- and post-stimulation), yielding percentage contributions per frequency band for each channel. The relative alpha increase was computed from these normalized percentages (Table 2, Table 3 and Table 4). Post-stimulation, alpha activity rose to approximately 50% of total occipital spectral power, delta contributions decreased markedly (from ~45% to ~15% in some channels), and other bands changed less consistently. This pattern indicates a frequency-selective redistribution of spectral energy toward the alpha-band rather than a broadband scaling of overall EEG amplitude.

## 4. Discussion

This study proposes an optimized neuromodulation device and examines the influence of low-field 10 Hz magnetic stimulation on human brain activity. It provides proof of concept in the field of neuromodulation, bridging engineering design and neurophysiological considerations. The study is a single-subject, no-sham design and these findings do not permit definitive causal inference regarding the clinical effectiveness of the device.

The original cylindrical coil was modified to follow a figure-of-eight configuration, as prior research underlined the significant influence coil geometry has on magnetic field focality and cortical targeting. Based on Maxwell–Faraday induction, current research shows that induced electric fields may result from a time-varying magnetic flux and enable transcranial magnetic stimulation in both high- and low-field regimes. To simulate this, we used the figure-of-eight coil as stimulus and a spherical head model as target. The simulation results pointed to more spatially confined magnetic gradients in the occipital area than in any other regions of the head. Although the anatomical model was simple, the results stay true to theoretical expectations and support the working hypothesis that spatial field shaping may lead to selective interactions with posterior alpha generators.

Electrophysiological tests were also performed to validate the proposed framework. Following 10 min of 10 Hz stimulation, occipital EEG recordings indicated a pronounced redistribution of spectral power toward the alpha-band. Post-stimulation alpha activity accounted for approximately half of total occipital spectral energy, with relative increases exceeding 140% across analyzed electrodes, while slow-wave dominance decreased significantly. Importantly, this pattern reflects a frequency-aligned redistribution rather than a global amplification of EEG power. Even though the stimulation waveform contained harmonic components inherent to its sawtooth shape, the dominant spectral component remained centered within the alpha range, following a frequency-based modulation scheme.

Our results are consistent with effects presented in frequency-aligned neuromodulation research, specifically transcranial alternating current stimulation and synchronized magnetic stimulation. Though weak-field neuromodulation remains debated, theoretical and experimental evidence shows that even electric fields below classical depolarization thresholds may influence brain activity if temporally aligned with intrinsic oscillations. Posterior alpha rhythms, generated by thalamocortical circuits, are especially sensitive to rhythmic perturbation. Thus, the shift toward alpha-band dominance may reflect a modulation of network state rather than a direct neuronal excitation.

These findings represent preliminary proof-of-principle evidence, demonstrating technical feasibility rather than definitive proof of causal neuromodulation as the single-subject design and absence of sham control prevent exclusion of alternative explanations.

Our current work presents several limitations. Most significantly, the absence of a sham control condition prevents definitive causal attribution of the observed alpha-band changes to LFMS. Occipital alpha activity is highly sensitive to nonspecific factors including mental relaxation over time, resting-state variability, time-dependent fluctuations, and expectation effects. While the observed pattern—spatial specificity (occipital-dominant), frequency specificity (an alpha increase with concurrent delta decrease rather than broadband amplification), and consistency with the targeted 10 Hz stimulation frequency—supports a stimulation-related mechanism, alternative explanations cannot be excluded. Additionally, the spherical head model did not integrate multilayer conductivity differences or cortical geometry, and the induced electric field magnitude was estimated rather than directly simulated. The present findings should thus be interpreted as preliminary proof-of-principle evidence consistent with frequency-aligned low-field neuromodulation rather than definitive proof of causal effects. Furthermore, this single-participant design precludes any inference regarding reproducibility, inter-individual variability, or population-level generalizability.

Future work will include anatomically realistic finite element modeling to estimate induced electric field strength and gradient, implement controlled experimental designs with statistical validation, and examine phase synchronization and functional connectivity metrics in addition to spectral power. Close-loop stimulation schemes and dose–response characterization may further clarify the interaction between engineered magnetic fields and oscillatory brain dynamics. Within this broader context, the study demonstrates the possibility of integrating electromagnetic field engineering with electrophysiological measurement to enable and explore frequency-tuned neuromodulation strategies.

## 5. Conclusions

Concisely, this study demonstrates that low-field magnetic stimulation delivered by a 10 Hz positive ramp sawtooth signal over the occipital cortex is associated with a significant redistribution of EEG spectral power towards the alpha-band.

The simulation analyses showed that by replacing the original cylindrical coil with a figure-of-eight configuration, the magnetic field focality and penetration were improved. These findings indicate the importance of coil geometry in the distribution of spatial field gradients. The simulation results were accompanied by electrophysiological measurements and analyses, showing a substantial relative increase in alpha activity following stimulation in both cases.

Observations from combined computational and experimental analyses provide preliminary proof-of-concept evidence suggesting that the cortical oscillatory activity can be influenced by frequency-aligned low-field magnetic stimulation.

By integrating electromagnetic field modeling with spectral EEG assessment, the foundation for a feasible workflow from engineering to physiology has been laid. However, a thorough strategy for investigating oscillation-targeted neuromodulation needs additional work, including quantitative electric field estimation, a more realistic modeling of the anatomical regions, and statistically controlled experimental validation.

## Figures and Tables

**Table 1:** The experimentally determined electrical parameters of the two coils.

Electrical Parameter	Original Cylindrical Coil	Figure-of-Eight Coil
Inductance *Ls*	434.8 μH	579.6 μH
Resistance *Rs*	2.24 Ω	2.37 Ω

**Table 2:** Power distribution before treatment.

Freq. Band/Channel	Delta	Theta	Alpha	Beta	Gamma
O1_LE	66.35	4.71	**20.12**	7.11	1.71
O2_LE	71.03	3.99	**17.12**	6.05	1.8
Oz_LE	70.58	4.67	**16.24**	6.72	1.8

**Table 3:** Power distribution after treatment.

Freq. Band/Channel	Delta	Theta	Alpha	Beta	Gamma
O1_LE	22.93	7.6	**49.53**	15.74	4.19
O2_LE	23.5	7.14	**50.47**	14.9	3.98
Oz_LE	25.75	7.1	**48.82**	14.96	3.36

**Table 4:** Relative variation (percentage) in power distribution along the brainwave bands.

Freq. Band/Channel	Delta	Theta	Alpha	Beta	Gamma
O1_LE	−65.44	61.36	**146.17**	121.38	145.03
O2_LE	−66.92	78.95	**194.8**	146.28	121.11
Oz_LE	−63.52	52.03	**200.62**	122.62	86.67

## Data Availability

The original contributions presented in this study are included in the article. Further inquiries can be directed to the corresponding authors.
